# Sample size estimation for randomised controlled trials with repeated assessment of patient-reported outcomes: what correlation between baseline and follow-up outcomes should we assume?

**DOI:** 10.1186/s13063-019-3671-2

**Published:** 2019-09-13

**Authors:** Stephen J. Walters, Richard M. Jacques, Inês Bonacho dos Anjos Henriques-Cadby, Jane Candlish, Nikki Totton, Mica Teo Shu Xian

**Affiliations:** School of Health and Related Research (ScHARR), University of Sheffield, 30 Regent Street, Sheffield, S1 4DA UK

**Keywords:** Sample size estimation, Review, Randomised controlled trials, Health Technology Assessment, Publicly funded, Correlations, ANCOVA, Patient-reported outcome measures

## Abstract

**Background:**

Patient-reported outcome measures (PROMs) are now frequently used in randomised controlled trials (RCTs) as primary endpoints. RCTs are longitudinal, and many have a baseline (PRE) assessment of the outcome and one or more post-randomisation assessments of outcome (POST). With such pre-test post-test RCT designs there are several ways of estimating the sample size and analysing the outcome data: analysis of post-randomisation treatment means (POST); analysis of mean changes from pre- to post-randomisation (CHANGE); analysis of covariance (ANCOVA).

Sample size estimation using the CHANGE and ANCOVA methods requires specification of the correlation between the baseline and follow-up measurements. Other parameters in the sample size estimation method being unchanged, an assumed correlation of 0.70 (between baseline and follow-up outcomes) means that we can halve the required sample size at the study design stage if we used an ANCOVA method compared to a comparison of POST treatment means method. So what correlation (between baseline and follow-up outcomes) should be assumed and used in the sample size calculation? The aim of this paper is to estimate the correlations between baseline and follow-up PROMs in RCTs.

**Methods:**

The Pearson correlation coefficients between the baseline and repeated PROM assessments from 20 RCTs (with 7173 participants at baseline) were calculated and summarised.

**Results:**

The 20 reviewed RCTs had sample sizes, at baseline, ranging from 49 to 2659 participants. The time points for the post-randomisation follow-up assessments ranged from 7 days to 24 months; 464 correlations, between baseline and follow-up, were estimated; the mean correlation was 0.50 (median 0.51; standard deviation 0.15; range − 0.13 to 0.91).

**Conclusions:**

There is a general consistency in the correlations between the repeated PROMs, with the majority being in the range of 0.4 to –0.6. The implications are that we can reduce the sample size in an RCT by 25% if we use an ANCOVA model, with a correlation of 0.50, for the design and analysis. There is a decline in correlation amongst more distant pairs of time points.

## Background

Patient-reported outcome measures (PROMs) are now frequently used in randomised controlled trials (RCTs) as primary endpoints. All RCTs are longitudinal, and many have a baseline, or pre-randomisation (PRE) assessment of the outcome, and one or more post-randomisation assessments of outcome (POST).

For such pre-test post-test RCT designs, using a continuous primary outcome, the sample size estimation and the analysis of the outcome can be done using one of the following methods:

1. Analysis of post-randomisation treatment means (POST)
2. Analysis of mean changes from pre- to post-randomisation (CHANGE)
3. Analysis of covariance (ANCOVA).

For brevity (and following Frison and Pocock’s nomenclature [1]), these methods will be referred to as POST, CHANGE and ANCOVA respectively.

Sample size calculations are now mandatory for many research protocols and are required to justify the size of clinical trials in papers before they will be accepted for publication by journals [2]. Thus, when an investigator is designing a study to compare the outcomes of an intervention, an essential step is the calculation of sample sizes that will allow a reasonable chance (power) of detecting a pre-determined difference (effect size) in the outcome variable, when the intervention is actually effective, at a given level of significance. Sample size is critically dependent on the type of summary measure, the proposed effect size and the method of calculating the test statistic [3]. For example, for a given power and significance level, the sample size is inversely proportional to the square of the effect size, so halving the effect size will quadruple the sample size. For simplicity, this paper will assume that we are interested in comparing the effectiveness (or superiority) of a new treatment compared to a standard treatment, at a single point in time post-randomisation.

### Sample size

In a two-group study with a Normally distributed outcome, comparing POST-randomisation mean outcomes between two groups, the number of subjects per group *n*_POST_ assuming equal sample sizes and equal standard deviations (SDs) per group for a two-sided significance level α and power 1 – β is [4]:

$$ {n}_{POST}\ \mathrm{per}\ \mathrm{group}=\frac{2{\sigma}^2{\left[{Z}_{1-\alpha /2}+{Z}_{1-\beta}\right]}^2}{\delta^2}, $$

where:

δ is the target or anticipated difference in mean outcomes between the two groups

σ is the SD of the outcome post-randomisation (which is assumed to be the same in both groups)

Z_1 – α/2_ and Z_1 – β_ are the appropriate values from the standard normal distribution for the 100 (1 – α/2) and 100 (1 – β) percentiles respectively.

Consider a two-group study with a Normally distributed outcome, with a single baseline and single post-randomisation assessment of outcomes. Comparing mean outcomes between two groups, adjusted for the baseline or pre-randomisation value of the outcome, using an ANCOVA model for the number of subjects per group *n*_ANCOVA_ (assuming equal sample sizes and equal SDs, at baseline and post-randomisation, per group) for a two-sided significance level α and power 1 – β is:

$$ {n}_{ANCOVA}\ \mathrm{per}\ \mathrm{group}=\frac{2{\sigma}^2{\left[{Z}_{1-\alpha /2}+{Z}_{1-\beta}\right]}^2}{\delta^2}\left\{1-{\rho}^2\right\}. $$

Here, ρ denotes the correlation between the baseline and post-randomisation outcomes and σ is the post-randomisation SD, which is assumed to be the same as the baseline SD [1, 5]. Machin et al. [5] refer to the (1 – ρ^2^) term as the ’design effect’ (DE).

In a two-group study with a Normally distributed outcome, comparing the mean change in outcomes (i.e. post-randomisation outcome – baseline) between two groups, the number of subjects per group *n*_CHANGE_ (assuming equal sample sizes and equal SDs, at baseline and post-randomisation, per group) for a two-sided significance level α and power 1 – β is:

$$ {n}_{CHANGE}\ \mathrm{per}\ \mathrm{group}=\frac{2{\sigma}^2{\left[{Z}_{1-\alpha /2}+{Z}_{1-\beta}\right]}^2}{\delta_c^2}\left\{2-2\rho \right\}. $$

Here, δ_c_ is the target or anticipated difference in mean change in outcomes between the two groups and σ is the post-randomisation SD that is assumed to be the same as the baseline SD. If the expected mean values of the baseline outcomes are the same in both groups, which is likely in an RCT, then δc is the same as δ.

**Fig. 1:**
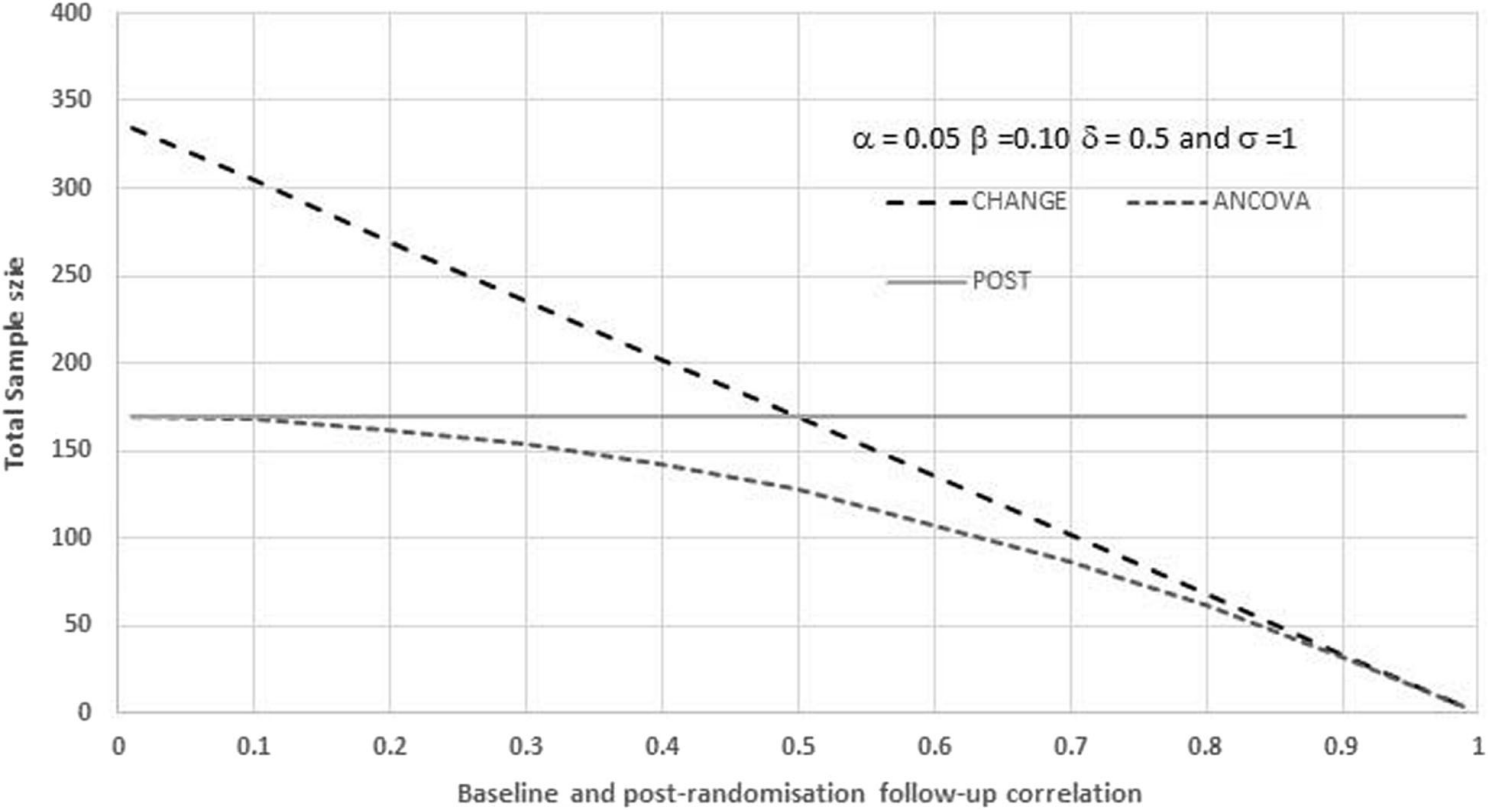
Relationship between the total sample size and the correlation between the baseline and post-randomisation outcomes for the three methods of sample size estimation (POST, CHANGE and ANCOVA)

Figure 1 shows the relationship between the total sample size and the correlation between the baseline and post-randomisation outcomes, for the three methods of sample size estimation (POST, CHANGE and ANCOVA) with a 5% two-sided significance level, 90% power, a target difference (a difference in post-treatment means or a difference in mean changes) of 0.50 and an SD of 1.0. Figure 1 shows how the total sample size is constant for POST irrespective of the baseline and post-randomisation follow-up correlation; the sample size declines as the correlation increases for ANCOVA and CHANGE; and that for correlations above 0.5 the sample size for ANCOVA is always the lowest and is less than or equal to the sample size for CHANGE.

### Example

The SELF study [6] was a multicentre, pragmatic, unblinded, parallel-group randomised control superiority trial designed to evaluate the clinical effectiveness of a self-managed single exercise programme versus usual physiotherapy treatment for rotator cuff tendinopathy (pain or weakness in the shoulder muscles). The intervention was a programme of self-managed exercise prescribed by a physiotherapist in relation to the most symptomatic shoulder movement. The control group received usual physiotherapy treatment. The primary outcome measure was the total score on the Shoulder Pain and Disability Index (SPADI) at 3 months post-randomisation. The SPADI Shoulder Score ranges from 0, being the best outcome (less disability), to 100 the worst (greater disability).

The original sample size calculation for the SELF trial assumed that a 10-point difference in the mean 3 months post-randomisation SPADI scores between the intervention and control groups would be regarded as a minimum clinical important difference (MCID). It assumed an SD of 24 points, a power of 80% and a (two-sided) significance level of 5%, meaning that using the POST sample size formula, 91 participants per group were required (182 in total). However, in light of new information from an external pilot study, the investigators undertook a sample size re-estimation (SSR) calculation, which was approved by the ethics committee. The new information related to a narrower estimate of population variance from an external pilot RCT (*n* = 24) of 16.8 points on the SPADI and, additionally, a correlation between baseline and 3 months SPADI scores of 0.5. Using the ANCOVA sample size formula, with an SD of 17 points; correlation between baseline and 3 months SPADI scores of 0.50, 80% power, 5% two-sided significance and a MCID (as before) of 10 points, it was estimated that 34 participants per group were required (68 in total). This contrasts with a sample size of 45 per group using the POST means formula with the revised SD of 17 points. Thus, with a correlation of 0.50 between baseline and follow-up, using the ANCOVA method for sample size estimation, we can reduce the sample size by approximately 25% (i.e. 1–0.5^2^) compared to the POST treatment means method.

Should the method of sample size estimation mirror the proposed method of statistical analysis (of the outcome data)? That is, if an ANCOVA model is likely to be used in the statistical analysis of the collected outcome data, should an ANCOVA method that allows for the correlation also be used in the sample size estimation method? And if so, what correlation (between baseline and follow-up outcomes) should be assumed and used in the sample size estimation? Other factors/parameters in the sample size estimation method being unchanged, an assumed correlation of 0.70 (between baseline and follow-up outcomes) means that we can halve the require sample size at the study design stage, if we used an ANCOVA method compared to a comparison of POST treatment means method. It is, however, paramount to assess how realistic a correlation of 0.50 or 0.70 between baseline and post-randomisation outcomes is, and to make evidence-based assumptions on these values, as an overestimated correlation could result in an underpowered study. The aim of this paper is to estimate the observed correlations between baseline and post-randomisation follow-up PROMs from a number of RCTs, bridging a gap in the evidence.

## Methods

### Data sources

This was a secondary analysis of RCTs with continuous patient-reported outcomes (both primary and secondary) undertaken in the School of Health and Related Research (ScHARR) at the University of Sheffield published between 1998 and 2019. Secondary ethics approval was gained through the University of Sheffield ScHARR Ethics Committee (Reference 024041).

### Statistical analysis

For each included trial, the correlation between baseline and post-randomisation outcomes was calculated using the Pearson correlation coefficient [7]. Given a set of *n* pairs of observations (*x*_1_, *y*_1_), (*x*_2_, *y*_2_), …, (*x*_*n*_, *y*_*n*_), with means $\overline{x}$ and $\overline{y}$ respectively, then the Pearson correlation coefficient *r* is given by:

$$ r=\frac{\sum \limits_{i=1}^n\left({y}_i-\overline{y}\right)\left({x}_i-\overline{x}\right)}{\sqrt{\sum \limits_{i=1}^n{\left({y}_i-\overline{y}\right)}^2\sum \limits_{i=1}^n{\left({x}_i-\overline{x}\right)}^2}} $$

with a standard error SE(*r*) = $SE(r)=\sqrt{\frac{1-{r}^2}{n-2}}$.

A variety of summary statistics for the baseline and post-randomisation correlations were calculated, including (1) the unweighted sample mean and median; (2) a weighted sample mean, using the fixed effect inverse variance method [4], and (3) a sample mean with allowance for clustering by trial derived from a multilevel mixed-effects linear model with a random effect for the trial using restricted maximum likelihood estimation (REML) [8]. The correlations were calculated overall and then split by trial, outcome and time point.

## Results

### Trials

Table 1 shows a summary of the 20 RCTs included in the analysis. Various outcome measures were used in the trials for both the primary and secondary outcomes. Table 2 provides a brief description of the outcome measures and how they were scaled. Three of the outcome measures, the Clinical Outcomes in Routine Evaluation - Outcome Measure (CORE-OM), Pelvic Organ Prolapse/Urinary Incontinence Sexual Questionnaire (PISQ-31) and SPADI, have a total score and various subscales: both were included in the analysis. The 20 included RCTs had sample sizes (at baseline) ranging from 49 to 2659 participants. The time points for the post-randomisation to follow-up assessments ranged from 7 days to 24 months. The maximum sample size for the baseline follow-up correlations ranged from 39 to 2659 participants. Four-hundred and sixty-four correlations between baseline and follow-up were estimated in the 20 trials. Table 1 shows, for example, that the Leg Ulcer trial (Trial 1) had 9 outcomes all assessed at 2 post-randomisation time points (3 and 12 months), giving a total of 2 × 9 = 18 correlations. The median number of outcomes per trial was 9 and ranged from 1 (in the 3Mg trial) to 15 (AIM-High, PLINY and IPSU). The median number of correlations calculated per trial was 16.5 and ranged from 1 (in the 3Mg trial) to 65 (in the DiPALS trial). The median number of post-randomisation follow-up time points across the 20 trials was 2.5 and ranged from 1 to 6.

**Table 1:** Summary of the 20 randomised controlled trials

	Trial name	Trial population	Age range (years)	Outcome measures	No. of outcomes	Time points post-randomisation	No. of time points	No. of correlations	Sample size at baseline	Max *N*^a^	Reference	Year of publication
1	Leg Ulcer	Leg ulcers	32 to 97	EQ-5D, SF-36	9	3, 12 months	2	18	233	200	[9]	1998
2	NAMEIT	Early severe rheumatoid arthritis	18 to 75	SF-36, SF-6D	9	2, 4, 6, 8, 10, 12 months	6	54	222	222	[10]	2000
3	Homeopathy for CFS	Chronic fatigue syndrome (CFS)	20 to 62	MFI	5	6 months	1	5	103	85	[11]	2004
4	Acupuncture	Low back pain	20 to 64	SF-36	8	3, 12, 24 months	3	24	239	217	[12]	2005
5	Knee Replacement	Osteoarthritis patients undergoing total knee replacement	51 to 92	SF-36, WOMAC	14	3 months	1	14	151	114	[13]	2005
6	FED	Older (aged ≥ 65) hospitalised patients with acute illness	65 to 93	Barthel, SF-36	9	1.5, 6 months	2	18	445	225	[14]	2006
7	AIM-High	Malignant melanoma	18 to 77	EORTC QLQ-C30	15	6, 12, 18, 24 months	4	60	444	392	[15]	2006
8	PoNDER	New mothers	18 to 45	CORE-OM, EPDS,^b^ SF-36, SF-6D	9	4.5, 10.5, 16.5 months	3	26	2659	2659	[16]	2009
9	COPD	Chronic obstructive pulmonary disease (COPD)	49 to 86	EQ-5D, SF-36, SF-6D	12	2, 6, 12, 18 months	4	48	238	172	[17]	2010
10	Corn Plasters	Foot corns	18 to 90	EQ-5D, EQ-5D VAS, VAS Pain	3	3, 6, 9, 12 months	4	12	201	182	[18]	2013
11	PLINY	Independently living older people (aged ≥ 75)	75 to 95	EQ-5D, EQ-5D VAS, GSES, ONS Well-being, PHQ-9, SF-36	15	6 months	1	15	157	56	[19]	2014
12	3Mg	Adults with acute severe asthma in the emergency department	16 to 88	EQ-5D	1	1 month	1	1	932	437	[20]	2014
13	SELF	Shoulder rotator cuff tendinopathy	23 to 83	SPADI	3	3, 6, 12 months	3	9	85	59	[6]	2016
14	BEADS	Post-stroke depression	31 to 97	EQ-5D, EQ-5D VAS, PHQ-9	3	6 months	1	3	49	39	[21]	2016
15	DiPALS	Amyotrophic lateral sclerosis (ALS)	23 to 83	EQ-5D, SAQLI, SF-36	13	2, 3, 6, 9, 12 months	5	65	74	55	[22]	2016
16	Lifestyle Matters	Independently living older people (aged ≥ 65)	65 to 92	EQ-5D, EQ-5D VAS, GSES, PHQ-9, SF-36	14	6, 24 months	2	28	288	262	[23]	2017
17	IPSU	Women with urinary incontinence and sexual dysfunction	21 to 70	EQ-5D, PISQ-31, SF-36	15	6 months	1	15	107	66	[24]	2018
18	POLAR	Lumbar radicular syndrome (LRS)	23 to 71	Back Pain VAS, EQ-5D, EQ-5D VAS, Leg Pain VAS, ODI	5	1.5, 3, 6 months	3	15	80	73	[25]	2018
19	PRACTICE	COPD	40 to 92	EQ-5D, EQ-5D VAS	2	0.25, 1, 3 months	3	6	55	42	[26]	2018
20	STEPWISE	Schizophrenia	18 to 71	B-IPQ, BPRS, EQ-5D, EQ-5D VAS, PHQ-9, RAND SF-36	14	3, 12 months	2	28	412	358	[27]	2018
								464	7173	5915		

^a^Max *N* is the maximum sample size for the baseline and post-randomisation follow-up correlations

^b^For the PoNDER trial the EPDS was measured at baseline and at 4.5 and 10.5 months post-randomisation

Abbreviations: *Barthel* Barthel Index for Activities of Daily Living (ADL), *B-IPQ* Brief Illness Perception Questionnaire, *BPRS* Brief Psychiatric Rating Scale, *CORE-OM* Clinical Outcomes in Routine Evaluation-Outcome Measure, *EORTC QLQ* European Organisation for Research and Treatment of Cancer Quality of Life Questionnaire, *EPDS* Edinburgh Postnatal Depression Scale, *EQ-5D* EuroQol Five Dimension, *GSES* General Self-Efficacy Scale, *MFI* Multidimensional Fatigue Inventory, *ODI* Oswestry Disability Index, *ONS* Office for National Statistics Well-being Questionnaire, *PHQ-9* Patient Health Questionnaire, *PISQ-31* Pelvic Organ Prolapse/Urinary Incontinence Sexual Questionnaire, *RAND SF-36* Research and Development 36-item Short Form Survey Instrument, *SAQLI* Sleep Apnea Quality of Life Index, *SF-36* Short Form (36 item) Health Survey, *SF-6D* Short Form Six Dimension, *SPADI* Shoulder Pain and Disability Index, *VAS* visual analogue scale, *WOMAC* Western Ontario and McMaster Universities Osteoarthritis Index

**Table 2:** Description of the outcome measures used in 20 randomised controlled trials and how they are scaled/scored

	Outcome name	Trials	Score range	Correlations	Reference
1	B-IPQ	1	0 to 10	2	[28]
2	Back Pain VAS	1	0 to 10	3	[29]
3	Barthel score	1	0 to 20	2	[30]
4	BPRS	1	0 to 126	2	[31]
5	CORE Life functioning dimension	1	0 to 4	3	[32]
6	CORE Risk/harm dimension	1	0 to 4	3	
7	CORE Problems/symptoms dimension	1	0 to 4	3	
8	CORE Subjective well-being dimension	1	0 to 4	3	
9	CORE total score	1	0 to 4	3	
10	EORTC Appetite Loss	1	0 to 100	4	[33]
11	EORTC Cognitive Functioning	1	0 to 100	4	
12	EORTC Constipation	1	0 to 100	4	
13	EORTC Diarrhoea	1	0 to 100	4	
14	EORTC Dyspnoea	1	0 to 100	4	
15	EORTC Emotional Functioning	1	0 to 100	4	
16	EORTC Fatigue	1	0 to 100	4	
17	EORTC Financial Difficulties	1	0 to 100	4	
18	EORTC Insomnia	1	0 to 100	4	
19	EORTC Nausea	1	0 to 100	4	
20	EORTC Pain	1	0 to 100	4	
21	EORTC Physical Functioning	1	0 to 100	4	
22	EORTC QoL	1	0 to 100	4	
23	EORTC Role Functioning	1	0 to 100	4	
24	EORTC Social Functioning	1	0 to 100	4	
25	EPDS	1	0 to 30	2	[34]
26	EQ-5D Utility score	12	−0.56 to 1.00	29	[35]
27	EQ-5D VAS	8	0 to 100	21	[36]
28	GSES	2	10 to 40	3	[37]
29	Leg Pain VAS	1	0 to 10	3	[29]
30	MFI General Fatigue	1	4 to 20	1	[38]
31	MFI Mental Fatigue	1	4 to 20	1	
32	MFI Physical Fatigue	1	4 to 20	1	
33	MFI Reduced Activity	1	4 to 20	1	
34	MFI Reduced Motivation	1	4 to 20	1	
35	ODI	1	0 to 100	3	[39]
36	ONS Well-Being	1	0 to 40	1	[40]
37	PHQ-9	4	0 to 27	6	[41]
38	PISQ-31 Behaviour/Emotion	1	0 to 61	1	[42]
39	PISQ-31 Partner-Related Factor	1	0 to 24	1	
40	PISQ-31 Physical Factor	1	0 to 40	1	
41	PISQ-31 total score	1	0 to 125	1	
42	SF-36 General Health	11	0 to 100	29	[43]
43	SF-36 Mental Health	11	1 to 100	29	
44	SF-36 Pain	11	1 to 100	29	
45	SF-36 Physical Functioning	11	1 to 100	29	
46	SF-36 Role-Emotional	11	1 to 100	29	
47	SF-36 Role-Physical	11	1 to 100	29	
48	SF-36 Social Functioning	11	1 to 100	29	
49	SF-36 Vitality	11	1 to 100	29	
50	SF-36 Health Change	1	1 to 100	2	
51	SF-36 Mental Component Summary	7	NBS	17	[44]
52	SF-36 Physical Component Summary	7	NBS	17	
53	SF-6D	4	0.29 to 1.00	14	[45]
54	Sleep Apnoea QoL	1	1 to 7	5	[46]
55	SPADI Disability	1	0 to 100	3	[47]
56	SPADI Pain	1	0 to 100	3	
57	SPADI total score	1	0 to 100	3	
58	VAS Pain	1	0 to 10	4	[48]
59	WOMAC Pain	1	0 to 20	1	[49]
60	WOMAC Physical Function	1	0 to 68	1	
61	WOMAC Stiffness	1	0 to 8	1	

*NBS* norm-based scoring: scores are standardised to have a mean of 50 and SD of 10

### Correlation

**Fig. 2:**
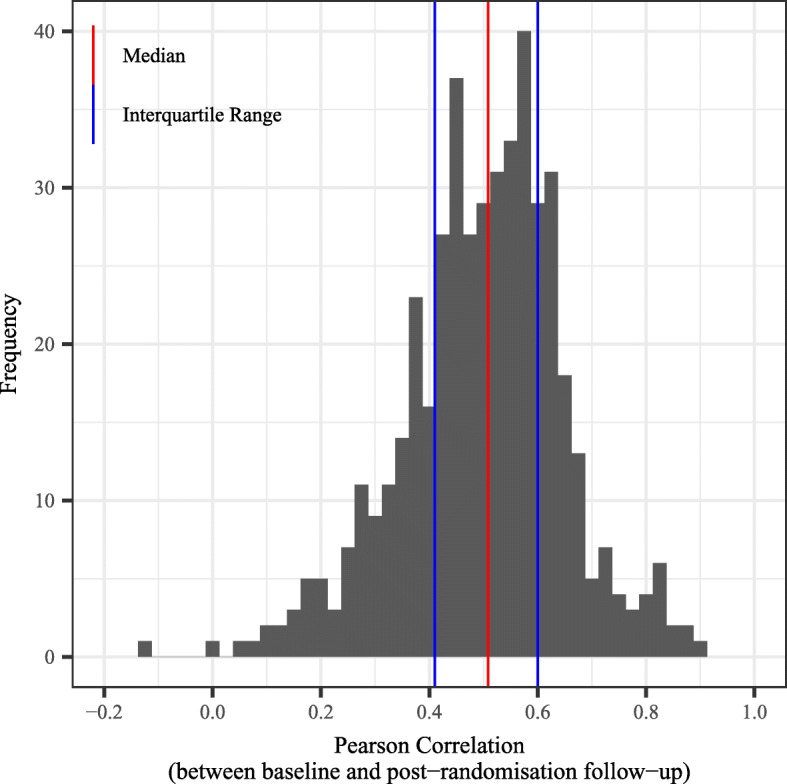
Histogram of *n* = 464 correlations with overall median, 25th and 75th percentiles

Figure 2 shows a histogram of the 464 estimated baseline to follow-up correlations. The histogram is reasonably symmetrical, and the overall mean correlation was 0.50 (median of 0.51). The baseline to follow-up correlations ranged from − 0.13 to 0.91 with an interquartile range of 0.41 to 0.60. Since the sample sizes for the trials varied from 49 to 2659 participants, a weighted estimate of the mean correlation, using the inverse variance method, was 0.51. Since the 464 correlation estimates were from 20 trials and the correlations were nested or clustered with trials, the estimated mean correlation after allowing for clustering by trial, using a multilevel mixed-effects linear regression model (with a random effect or intercept for the trial), was 0.49 (95% confidence interval [CI] 0.45 to 0.53). These other summary estimates were very similar to the simple unweighted mean value of 0.50.

**Fig. 3:**
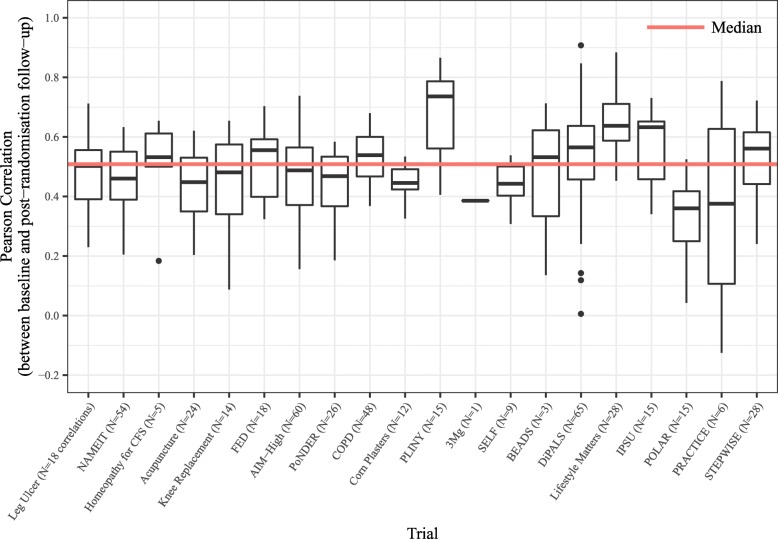
Box and whisker plot of (*n* = 464) correlations by trial

Table 3 shows the baseline to post-randomisation follow-up correlations aggregated by trial. The largest average correlations per trial showed a mean of 0.67 observed in the PLINY trial; the lowest average correlations were observed in the POLAR trial. The trial with the widest range of correlations was the PRACTICE trial. Figure 3 shows a box and whisker plot of how the observed baseline to follow-up correlations varied across the 20 RCTs along with the overall median correlation. There was considerable intertrial variation in the correlations, and it should be noted that some of the trials had less than or equal to six baseline to follow-up correlations estimated (3Mg [*N* = 1 outcome and correlation], BEADS [*N* = 3], Homeopathy [*N* = 5] and PRACTICE [*N* = 6]).

**Table 3:** Baseline to post-randomisation follow-up correlations by trial

Trial name	Pearson baseline to post-randomisation follow-up correlation					
	Mean	Median	No. of correlations	SD	Minimum	Maximum
Leg Ulcer	0.48	0.5	*N* = 18	0.13	0.23	0.71
NAMEIT	0.46	0.46	*N* = 54	0.1	0.21	0.63
Homeopathy for CFS	0.5	0.53	*N* = 5	0.19	0.18	0.65
Acupuncture	0.44	0.45	*N* = 24	0.12	0.2	0.62
Knee Replacement	0.45	0.48	*N* = 14	0.16	0.09	0.65
FED	0.5	0.56	*N* = 18	0.12	0.32	0.7
AIM-High	0.46	0.49	*N* = 60	0.14	0.16	0.74
PoNDER	0.44	0.47	*N* = 26	0.11	0.19	0.58
COPD	0.53	0.54	*N* = 48	0.08	0.37	0.68
Corn Plaster	0.45	0.45	*N* = 12	0.06	0.33	0.53
PLINY	0.67	0.74	*N* = 15	0.15	0.41	0.87
3Mg	0.39	0.39	*N* = 1	-	0.39	0.39
SELF	0.44	0.44	*N* = 9	0.07	0.31	0.54
BEADS	0.46	0.53	*N* = 3	0.3	0.14	0.71
DiPALS	0.54	0.57	*N* = 65	0.18	0.01	0.91
Lifestyle Matters	0.66	0.64	*N* = 28	0.11	0.45	0.88
IPSU	0.57	0.63	*N* = 15	0.13	0.34	0.73
POLAR	0.32	0.36	*N* = 15	0.14	0.04	0.53
PRACTICE	0.36	0.38	*N* = 6	0.36	−0.13	0.79
STEPWISE	0.53	0.56	*N* = 28	0.13	0.24	0.72
Total^a^	0.5	0.51	*N* = 464	0.15	−0.13	0.91

^a^The summary statistics for the total row are calculated from 464 correlations

**Fig. 4:**
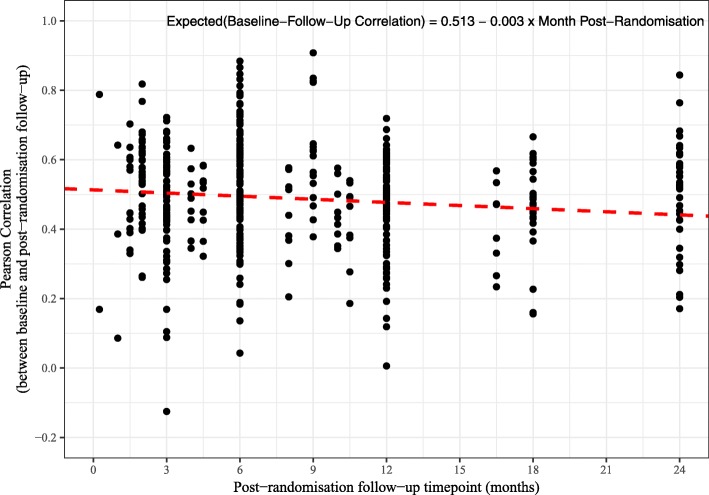
Scatter plot of correlations against post-randomisation follow-up time point with regression line (464 correlations from 20 trials)

**Fig. 5:**
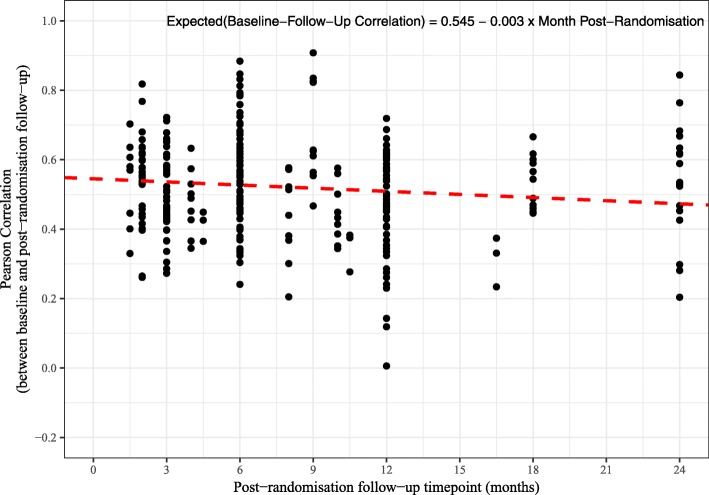
Scatter plot of correlations against post-randomisation follow-up time point with regression line, SF-36 outcomes (282 correlations from 12 trials)

**Fig. 6:**
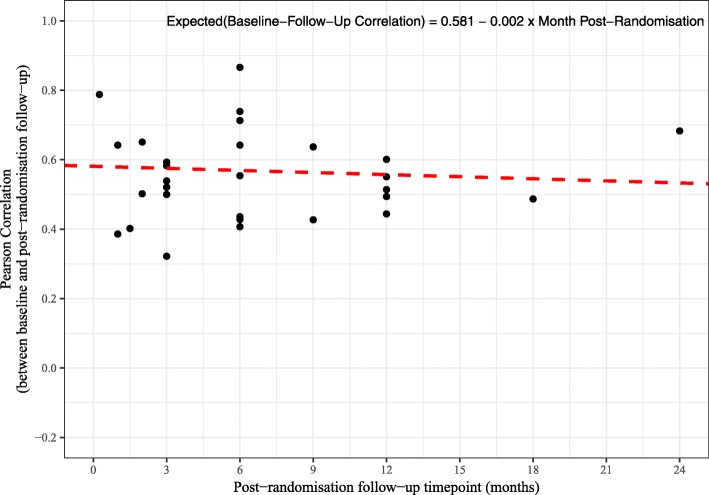
Scatter plot of correlations against post-randomisation follow-up time point with regression line, EQ-5D Utility outcome (29 correlations from 12 trials)

The time points for the post-randomisation follow-up assessments ranged from 7 days to 24 months. Table 4 shows the baseline to post-randomisation follow-up correlations by post-randomisation follow-up time point. Figure 4 shows a scatter plot of the baseline to follow-up correlations by post-randomisation follow-up time point for the 464 correlations from the 20 trials. Although it is not obvious from the scatter plot, a multilevel mixed-effects linear regression model (with a random intercept for the trial) suggests a small decline in the baseline to post-randomisation follow-up correlations the further the time points are apart. The estimated regression coefficient from the model was − 0.003 (95% CI − 0.006 to − 0.001; *P* = 0.005). This implies that for every unit or 1-month increase in the time from baseline to the post-randomisation follow-up the correlation declines by 0.003 point. Figures 5 and 6 show how the correlations change over time for the Short Form Health Survey (SF-36) outcomes (282 correlations and 12 trials) and the EuroQol five dimension scale (EQ-5D) Utility score outcome (29 correlations and 12 trials). A similar pattern to the overall pattern is observed for these specific outcomes with a small decline (0.003 for the SF-36 and 0.002 for the EQ-5D) in baseline to follow-up correlations over time.

**Table 4:** Baseline to post-randomisation follow-up correlations by post-randomisation follow-up time point

Post-randomisation follow-up time point (months)	Pearson baseline to post-randomisation follow-up correlation					
	Mean	Median	SD	Minimum	Maximum	No. of correlations
0.25	0.48	0.48	0.44	0.17	0.79	2
1	0.37	0.39	0.28	0.09	0.64	3
1.5	0.49	0.45	0.12	0.33	0.70	14
2	0.55	0.56	0.12	0.26	0.82	34
3	0.48	0.50	0.15	−0.13	0.72	71
4	0.48	0.49	0.09	0.35	0.63	9
4.5	0.48	0.52	0.09	0.32	0.58	9
6	0.54	0.55	0.16	0.04	0.88	121
8	0.43	0.44	0.13	0.21	0.58	9
9	0.62	0.62	0.16	0.38	0.91	16
10	0.45	0.43	0.08	0.34	0.58	9
10.5	0.42	0.47	0.12	0.19	0.54	9
12	0.46	0.48	0.14	0.01	0.72	86
16.5	0.41	0.42	0.12	0.23	0.57	8
18	0.47	0.49	0.13	0.16	0.67	27
24	0.51	0.53	0.15	0.17	0.84	37

Table 5 shows the baseline to post-randomisation correlations by outcome. The SF-36 was the most popular outcome and used in 12 out of the 20 trials. The correlations for SF-36 outcomes and its various dimensions (12 trials and *n* = 282 correlations) showed a mean of 0.51 (median 0.53), range 0.06 to 0.91. The second most popular outcome was the EQ-5D, which was used in 12 of the trials as well. Correlations for EQ-5D outcomes only (12 trials and *n* = 50 correlations) showed a mean of 0.49 (median 0.51), range − 0.13 to 0 87. Three of the outcome measures, the CORE-OM, PISQ-31 and SPADI, in Table 5 have a total score and various subscales. There was no clear pattern in the correlations and no reliable evidence that the total scale score correlated more highly than an individual subscale score.

**Table 5:** Baseline to post-randomisation follow-up correlations by outcome

Outcome	Pearson baseline to post-randomisation follow-up correlation					
	Mean	Median	SD	Minimum	Maximum	No. of correlations
B-IPQ	0.6	0.6	0	0.6	0.61	2
BACK PAIN	0.24	0.19	0.11	0.17	0.37	3
Barthel Score	0.53	0.53	0.1	0.45	0.6	2
BPRS	0.57	0.57	0.12	0.49	0.65	2
CORE Functioning Dimension	0.56	0.57	0.02	0.54	0.58	3
CORE Risk Dimension	0.26	0.27	0.07	0.19	0.32	3
CORE Symptoms Dimension	0.5	0.49	0.03	0.47	0.54	3
CORE Total Score	0.55	0.53	0.03	0.53	0.58	3
CORE Well Being Dimension	0.5	0.49	0.03	0.47	0.53	3
EORTC Appetite Loss	0.29	0.3	0.09	0.21	0.37	4
EORTC Cognitive Functioning	0.49	0.49	0.08	0.4	0.59	4
EORTC Constipation	0.39	0.4	0.08	0.3	0.48	4
EORTC Diarrhoea	0.25	0.27	0.07	0.16	0.32	4
EORTC Dyspnoea	0.43	0.44	0.06	0.35	0.49	4
EORTC Emotional Functioning	0.51	0.5	0.06	0.46	0.6	4
EORTC Fatigue	0.56	0.56	0.06	0.49	0.63	4
EORTC Financial Difficulties	0.63	0.62	0.07	0.56	0.74	4
EORTC Insomnia	0.44	0.49	0.12	0.26	0.52	4
EORTC Nausea	0.21	0.18	0.06	0.16	0.3	4
EORTC Pain	0.48	0.48	0.08	0.39	0.58	4
EORTC Physical Functioning	0.59	0.58	0.07	0.52	0.68	4
EORTC QoL	0.55	0.57	0.08	0.44	0.61	4
EORTC Role Functioning	0.54	0.54	0.04	0.5	0.59	4
EORTC Social Functioning	0.51	0.5	0.06	0.43	0.59	4
EPDS	0.49	0.49	0.04	0.47	0.52	2
EQ-5D Utility Score	0.55	0.54	0.13	0.32	0.87	29
EQ-5D VAS	0.41	0.46	0.2	−0.13	0.67	21
GSES	0.52	0.56	0.08	0.44	0.58	3
LEG PAIN	0.16	0.11	0.16	0.04	0.34	3
MFI General Fatigue	0.18	0.18	NA	0.18	0.18	1
MFI Mental Fatigue	0.53	0.53	NA	0.53	0.53	1
MFI Physical Fatigue	0.5	0.5	NA	0.5	0.5	1
MFI Reduced Activity	0.65	0.65	NA	0.65	0.65	1
MFI Reduced Motivation	0.61	0.61	NA	0.61	0.61	1
ODI	0.36	0.36	0.05	0.31	0.41	3
ONS Well-Being	0.62	0.62	NA	0.62	0.62	1
PHQ9	0.66	0.66	0.08	0.53	0.76	6
PISQ-31 Behaviour/Emotion	0.73	0.73	NA	0.73	0.73	1
PISQ-31 Partner Related Factor	0.63	0.63	NA	0.63	0.63	1
PISQ-31 Physical Factor	0.35	0.35	NA	0.35	0.35	1
PISQ-31 Total Score	0.62	0.62	NA	0.62	0.62	1
SF-36 General Health	0.6	0.58	0.08	0.49	0.79	29
SF-36 Mental Component Summary	0.54	0.55	0.12	0.33	0.79	17
SF-36 Mental Health	0.57	0.57	0.11	0.37	0.83	27
SF-36 Pain	0.49	0.51	0.13	0.2	0.71	29
SF-36 Physical Component Summary	0.56	0.6	0.21	0.14	0.84	17
SF-36 Physical Functioning	0.64	0.63	0.17	0.01	0.91	29
SF-36 Role-Emotional	0.42	0.43	0.11	0.12	0.68	31
SF-36 Role-Physical	0.39	0.35	0.12	0.21	0.67	29
SF-36 Social Functioning	0.44	0.45	0.1	0.24	0.63	29
SF-36 Vitality	0.55	0.53	0.1	0.43	0.82	29
SF-36 Health Change	0.32	0.32	0.11	0.24	0.4	2
SF-6D	0.5	0.48	0.09	0.37	0.64	14
Sleep Apnoea QoL	0.56	0.6	0.12	0.35	0.65	5
SPADI	0.47	0.47	0.03	0.44	0.5	3
SPADI Disability	0.49	0.51	0.06	0.43	0.54	3
SPADI Pain	0.36	0.38	0.05	0.31	0.4	3
VAS Pain	0.41	0.41	0.07	0.33	0.48	4
WOMAC Pain	0.26	0.26	NA	0.26	0.26	1
WOMAC Physical Function	0.46	0.46	NA	0.46	0.46	1
WOMAC Stiffness	0.09	0.09	NA	0.09	0.09	1

## Discussion

The 20 reviewed RCTs had sample sizes, at baseline, ranging from 49 to 2659 participants. The time points for the post-randomisation follow-up assessments ranged from 7 days to 24 months; 464 correlations between baseline and follow-up were estimated; the mean correlation was 0.50 (median 0.51; SD 0.15; range − 0.13 to 0.91).

The 20 RCTs included in this study were a convenience sample of trials and data and may not be representative of the population of all trials with PROMs. However, they include a wide range of populations and disease areas, a variety of different interventions and outcomes that are not untypical of other published trials. We also reviewed detailed reports of 181 RCTs published in the National Institute for Health Research (NIHR) Health Technology Assessment (HTA) journal from 2004 to the end of July 2017 and found 11 NIHR HTA reports (and 12 outcomes) that had a sample size calculation based on the ANCOVA model [50]. For these 12 outcomes the mean baseline to follow-up correlation that was assumed and used in the subsequent sample size calculation was 0.49 (SD 0.09) and ranged from 0.31 to 0.60. Thus, our results, with a mean correlation of 0.50, are consistent with correlations used and published in the NIHR HTA journal.

We observed a small decline in baseline to follow-up correlations over time of − 0.003 per month. That is, for every unit or 1-month increase in the time from baseline to the post-randomisation follow-up, the correlation declines by 0.003 point. Frison and Pocock [1] also report a slight decline in correlation amongst more distant pairs of time points post-randomisation, with the estimated slope being − 0.009 per month apart. So our results are also consistent with a slight decline.

It is important to make maximum use of the information available from other related studies or extrapolation from other unrelated studies. The more precise the information, the better we can design the trial. We would recommend that researchers planning a study with PROMs as the primary outcome pay careful attention to any evidence on the validity and frequency distribution of the PROM and its dimensions.

Strictly speaking, our results and conclusions only apply to the study population and the outcome measures used in the 20 RCTs. Further empirical work is required to see whether these results hold true for other outcomes, populations and interventions. However, the PROMs in this paper share many features in common with other PROM outcomes, i.e. multidimensional, ordinal or discrete response categories with upper and lower bounds, and skewed distributions; therefore, we see no theoretical reasons why these results and conclusions may not be appropriate for other PROMs.

Throughout this paper, we only considered the situation where a single dimension of the PROM is used at a single endpoint. Sometimes there is more than one endpoint of interest; PROMs are typically multidimensional (e.g. the SF-36 has eight dimensions). If one of these dimensions is regarded as more important than the others, it can be named as the primary endpoint and the sample size estimates calculated accordingly. The remainder should be consigned to exploratory analyses or descriptions only.

We have also assumed a rather simple form of the alternative hypothesis that the new treatment/intervention would improve patient-reported outcomes compared to the control/standard therapy. This form of hypothesis (superiority versus equivalence) may be more complicated than actually presented. However, the assumption of a simple form of the alternative hypothesis—that the new treatment/intervention would improve outcomes compared to the control/standard therapy—is not unrealistic for most superiority trials and is frequently used for other clinical outcomes. Walters gives a more comprehensive discussion of multiple endpoints and suggests several methods for analysing PROMs [4].

Overall, 5 of the 464 observed correlations were small (less than 0.10). Two of these small correlations came from the PRACTICE trial [26]. In this trial (PRACTICE) we observed a negative correlation of − 0.13 (*n* = 36 participants) between the baseline and 3 months follow-up post-randomisation time point for the EQ-5D visual analogue scale (VAS) and 0.09 (*n* = 42 participants) between the baseline and 1 month follow-up. The correlations were based on small sample sizes (*n* = 36 and 42), and examination of the scatter plots suggested no outlying values and a random scatter. The EQ-5D VAS outcome asks respondents to rate their health today on a 0 (the worst health you can imagine) to 100 (best health you can imagine) visual analogue scale. It may be that there genuinely is no correlation in the population (of chronic obstructive pulmonary disease [COPD] patients) with this outcome.

We calculated several summary correlations to allow for clustering of the outcomes by trial and the variance or standard error of the correlation estimate. The overall summary correlation for the 464 correlations was robust to the summary measure (mean, median, weighted mean, clustered mean) and was around 0.50.

Clifton and Clifton [51] comment that baseline imbalance may occur in RCTs and that ANCOVA should be used to adjust for baseline in the analysis. Clifton et al. [52] also point out the following theoretical assumptions for using the ANCOVA method for sample size estimation: (1) the pairs of baseline and post-randomisation outcomes follow a bivariate normal distribution; (2) the values of the baseline to post-randomisation follow-up, *r*, are the same in both groups; (3) the variances or SDs of the outcomes are the same in both groups. However, ANCOVA is known to be robust to departures from the assumptions of Normality. The work of Heeren and D’Agostino [53]and Sullivan and D’Agostino [54] supports the robustness of the two independent samples *t* test and ANCOVA when applied to three-, four- and five-point ordinal scaled data using assigned scores (like PROMs), in sample sizes as small as 20 subjects per group.

## Conclusions

There is a general consistency in the correlations between the baseline and follow-up PROMs, with the majority being in the range from 0.4 to 0.6. The implications are that we can reduce the sample size in an RCT by 25% if we use an ANCOVA model, with a correlation of 0.50, for the design and analysis. When allowing for the correlation between baseline and follow-up outcome in the sample size calculation, it is preferable to be conservative and use existing data that are relevant to your outcome and your population if they are available. Secondly, be wary of having an ’automatic’ rule of adjusting your required sample size downwards by 25% just because you have a baseline assessment.

There is a slight decline in correlation between baseline and more distant post-randomisation follow-up time points. Finally, we would stress the importance of a sample size calculation (with all its attendant assumptions) and also stress that any such estimate is better than no sample size calculation at all, particularly in a trial protocol [55, 56]. The mere fact of calculation of a sample size means that a number of fundamental issues have been considered: what is the main outcome variable, what is a clinically important effect, and how is it measured? The investigator is also likely to have specified the method and frequency of data analysis. Thus, protocols that are explicit about sample size are easier to evaluate in terms of scientific quality and the likelihood of achieving objectives.

## Data Availability

The data set is available on request from the corresponding author at s.j.walters@sheffield.ac.uk.

## Abbreviations

ANCOVA: Analysis of covariance
COPD: Chronic obstructive pulmonary disease
DE: Design effect
HTA: Health Technology Assessment
MCID: Minimum clinical important difference
NIHR: National Institute for Health Research
PROM: Patient reported outcome measure
RCT: Randomised controlled trial
ScHARR: School of Health and Related Research
SPADI: Shoulder Pain & Disability Index
SSR: Sample size re-estimation
