# Correction to: Sample size estimation for randomised controlled trials with repeated assessment of patient-reported outcomes: what correlation between baseline and follow-up outcomes should we assume?

**DOI:** 10.1186/s13063-019-3732-6

**Published:** 2019-10-28

**Authors:** Stephen J. Walters, Richard M. Jacques, Inês Bonacho dos Anjos Henriques-Cadby, Jane Candlish, Nikki Totton, Mica Teo Shu Xian

**Affiliations:** School of Health and Related Research (ScHARR), University of Sheffield, 30 Regent Street, Sheffield, S1 4DA UK

**Correction to: Trials (2019) 20: 566**

**https://doi.org/10.1186/s13063-019-3671-2**

Following publication of the original article [1], we have been notified that one of an error in the Conclusions section of the Abstract.

The range of correlations should read “0.4 to **0.6**” instead of “0.4 to **-0.6**” in the phrase “There is a general consistency in the correlations between the repeated PROMs, with the majority 31 being in the range of 0.4 to 0.6.”

The original article has been corrected.

## References

[1] Walters SJ (2019). Sample size estimation for randomised controlled trials with repeated assessment of patient-reported outcomes: what correlation between baseline and follow-up outcomes should we assume?. Trials.

